# Task shifting in maternal and newborn care: a non-inferiority study examining delegation of antenatal counseling to lay nurse aides supported by job aids in Benin

**DOI:** 10.1186/1748-5908-6-2

**Published:** 2011-01-06

**Authors:** Larissa Jennings, André Sourou Yebadokpo, Jean Affo, Marthe Agbogbe, Aguima Tankoano

**Affiliations:** 1 USAID Health Care Improvement Project, University Research Co., LLC, Wisconsin Boulevard, Bethesda, MD, USA; 2 Department of Population, Family, and Reproductive Health, Johns Hopkins Bloomberg School of Public Health, Wolfe Street, Baltimore, MD, USA; 3 Integrated Family Health Project, University Research Co., LLC, BP 420, Bohicon, Benin

## Abstract

**Background:**

Shifting the role of counseling to less skilled workers may improve efficiency and coverage of health services, but evidence is needed on the impact of substitution on quality of care. This research explored the influence of delegating maternal and newborn counseling responsibilities to clinic-based lay nurse aides on the quality of counseling provided as part of a task shifting initiative to expand their role.

**Methods:**

Nurse-midwives and lay nurse aides in seven public maternities were trained to use job aids to improve counseling in maternal and newborn care. Quality of counseling and maternal knowledge were assessed using direct observation of antenatal consultations and patient exit interviews. Both provider types were interviewed to examine perceptions regarding the task shift. To compare provider performance levels, non-inferiority analyses were conducted where non-inferiority was demonstrated if the lower confidence limit of the performance difference did not exceed a margin of 10 percentage points.

**Results:**

Mean percent of recommended messages provided by lay nurse aides was non-inferior to counseling by nurse-midwives in adjusted analyses for birth preparedness (β = -0.0, 95% CI: -9.0, 9.1), danger sign recognition (β = 4.7, 95% CI: -5.1, 14.6), and clean delivery (β = 1.4, 95% CI: -9.4, 12.3). Lay nurse aides demonstrated superior performance for communication on general prenatal care (β = 15.7, 95% CI: 7.0, 24.4), although non-inferiority was not achieved for newborn care counseling (β = -7.3, 95% CI: -23.1, 8.4). The proportion of women with correct knowledge was significantly higher among those counseled by lay nurse aides as compared to nurse-midwives in general prenatal care (β = 23.8, 95% CI: 15.7, 32.0), birth preparedness (β = 12.7, 95% CI: 5.2, 20.1), and danger sign recognition (β = 8.6, 95% CI: 3.3, 13.9). Both cadres had positive opinions regarding task shifting, although several preferred 'task sharing' over full delegation.

**Conclusions:**

Lay nurse aides can provide effective antenatal counseling in maternal and newborn care in facility-based settings, provided they receive adequate training and support. Efforts are needed to improve management of human resources to ensure that effective mechanisms for regulating and financing task shifting are sustained.

## Background

Task shifting refers to the delegation of non-technical tasks traditionally held by professional workers to workers with lower qualifications [1]. Recent years have seen growing interest in the effectiveness of task shifting as a strategy for targeting expanding health care demands in settings with shortages of qualified health personnel. Task shifting is often introduced to enable professional workers to focus on more technical, life-saving roles and to expand coverage of effective interventions in areas with limited health personnel. While task shifting does not increase the number of qualified staff, delegating roles can mitigate a health system's dependence on highly skilled individuals for specific services [1].

Although the term is relatively new in the global health context, task shifting has been used for many years and in several countries. Numerous studies provide evidence across health-related areas on task shifting between non-physician clinicians and nurses or midwives [2-7]. However, these studies have often been based in developed countries, and past research in developing countries had predominantly focused of skilled cadre, such as comparing surgical technicians and doctors [8], medical assistants and nurses [9], or physicians and other professional staff [1,10,11]. More recent analyses have examined the effect of delegating tasks to nurses and lay health workers in HIV-related resource-poor settings [12-14]. There is also broad consensus on the deployment of community health workers to increase coverage of key services [15].

However, given the magnitude of current health crises, addressing human resource needs through task shifting remains neglected [16], and there is a dearth of literature on the comparative effectiveness of task shifting from specialized workers to lay providers [17,18]. Because many African countries have substantial shortages of skilled personnel, renewed interest in task shifting has grown, particularly in light of the current HIV and AIDS human resources crisis and in recognition that task shifting may be used to improve other health services [17]. For example, maternal, infant, and child mortality in many African contexts has been attributed to aggregate shortages of skilled providers -- highlighting opportunities where this approach can be explored [19-21]. In the context of counseling, this may mean that less skilled workers assume time-intensive counseling tasks to enable nurses or midwives to engage in higher-impact clinical services.

Task shifting has been praised on several fronts given its potential to improve the skill mix of teams [16,22], to lower costs for training and remuneration [23], to shift health care to cadres that are better retained [24], and support retention of existing cadres by reducing burnout from inefficient care processes [25]. Task shifting is welcomed for its potential to bring about more efficient use of health personnel while diverging from efforts that have previously failed, such as government post assignments or extensive medical training [16,22]. Rather, the approach emphasizes inclusion and, in some cases, development of a lower level cadre to assume tasks they are able to do. Yet, there can be resistance by higher cadres given perceived lessening of hierarchal structures [26], loss of earnings (where remuneration includes fee for services), and the additional supervisory responsibilities that more skilled staff must assume [25,27]. Research has shown that lay health workers who undertake specific training with clearly defined responsibilities can complement and support services provided by more skilled health workers [1,15,23,28], but questions remain on how services are coordinated [18] and the impact of substitution on the quality of care [25,29]. Evidence is needed likewise on the extent of support necessary for lower cadre workers to achieve high performance.

The World Health Organization (WHO) recently released guidelines on task shifting which emphasize the need to ensure that approaches are adopted as part of a broader strategy of health systems strengthening that includes mechanisms and research to make certain quality of care is not compromised [30,31]. To address this issue, we examined whether lay nurse aides supported by counseling job aids can provide communication to pregnant women in maternal and newborn care at similar (or better) performance levels than nurse-midwives who usually undertake this role. Specifically, the study aimed to determine if services provided by lay nurse aides were not 'significantly better,' that they were 'at least to the same standard' as those provided by nurse-midwives, with potential gains in other health care measures [2]. The study also documented the opinions and attitudes of both cadres, which has rarely been done in a developing country context. A recent literature review identified one study examining opinions of health personnel on clinical task delegation [32] in addition to a quality assessment of task shifting in an African country [33]. However, these studies involved only skilled providers or were not specifically related to maternal and newborn care. It is hoped that findings from this research will inform future task shifting approaches and policies to promote the health and survival of mothers and newborns.

## Methods

### Study design and context

This study used a non-inferiority quasi-experimental design. A non-inferiority study was used because the quality of communication by lay nurse aides was not required to be significantly better than that of nurse-midwives, but it was necessary that the quality of communication be at least to the same standard [34]. Seven public health maternities in Zou/Collines, Benin participated in the study (one zonal hospital and six commune health centers) in which nurse-midwives and lay nurse aides were trained to use a set of pictorial counseling job aids to improve quality of maternal and newborn care counseling to pregnant women. Thus, lay nurse aides assisted by job aids were compared to nurse-midwives using identical performance supports.

Antenatal care is traditionally conducted by trained nurse-midwives who provide antenatal clinical and communication services. Nurses and midwives in Benin undergo three years of standardized, government-accredited training in obstetric-gynecology and internal medicine. They are employees of the government with an average monthly salary of approximately $118 USD. Traditional roles of nurse-midwives consist of vital signs measurement, counseling, physical examination, and medication dispensation in antenatal care as well as management of deliveries and obstetrical complications. Lay nurse aides are also government employees with an average monthly salary of approximately $59 USD. They receive no formal education but rather are trained on-site by nurse-midwives to assist in tasks such as directing patient flow, taking vital signs, recording height and weight, record keeping, and cleaning. In the absence of a skilled provider, lay nurse aides may also informally provide other clinical services, although they are not trained to do so. To this extent, responsibilities sometimes overlap. The number of lay nurse aides often approximates that of nurse-midwives, although health facilities may have shortages of both cadres.

We tested the influence of shifting the role of counseling to lay nurse aides on quality of counseling and maternal knowledge. Data were collected two weeks prior to the counseling task shift among women counseled by nurse-midwives (standard group) and following the task shift among women counseled by lay nurse aides (comparison group).

### Sample selection

A non-inferiority design examines whether a comparison is no worse or 'non-inferior' to a standard group. Because exact equivalence cannot be determined [35,36], a margin of non-inferiority of 10% was set for the maximum allowable difference in indicators on quality of communication provided to women counseled by nurse-midwives as compared to lay nurse aides. The determination of the margin of non-inferiority was based on what was considered a clinically significant level of performance as well as study feasibility. Two hundred pregnant women were enrolled in each group to allow detection of performance no worse than 10% with 80% power at the 5% significance level and a standard design effect of 2.0.

Seven public health centers with sufficient patient loads were selected to achieve the target sample size and increase the generalizability of findings among health centers with slightly varying service and patient characteristics. These facilities were operated by the Benin Ministry of Health and purposively selected within the Zou/Collines region among sites supported by the Integrated Health Project (PISAF-Projet Intégré de Santé Familiale) under the management of University Research Co., LLC (URC). Participant eligibility criteria included being pregnant and presenting at the health facility for antenatal consultation during the data collection period, willingness to be interviewed after the clinic visit, and (when applicable) willingness to be counseled by a lay nurse aide with training identical to that of nurse-midwives in use of the counseling materials. Using systematic sampling, eligible women were approached while waiting for consultation, given information regarding the purpose of the study, and invited to participate. Participation from site managers and providers was obtained prior to the start of the study.

### Task shifting intervention

**Figure 1:**
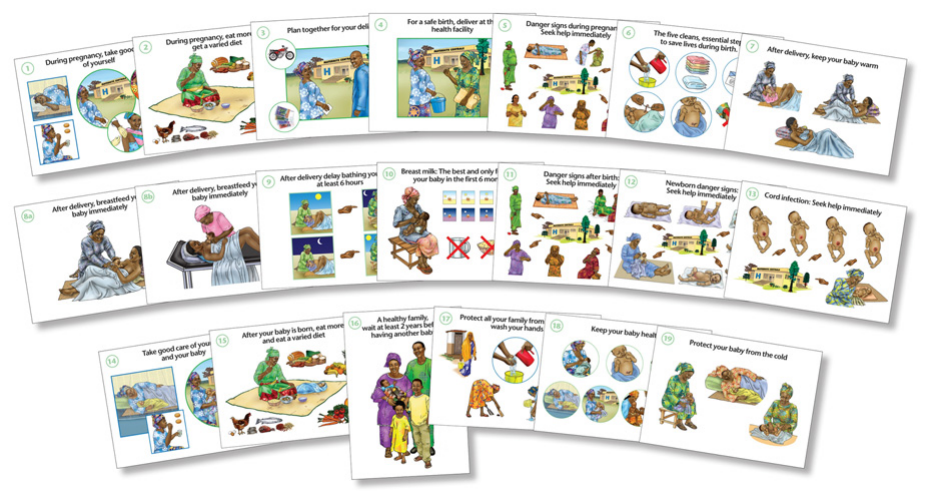
**Counseling job aids used for communication regarding pregnancy care, birth preparedness, danger signs, clean delivery, and newborn care**. Actual size 8 × 11 (A1 sheet)

This study was part of a larger study to evaluate the effectiveness of using a set of pictorial counseling cards to improve quality of antenatal counseling among nurse-midwives. Previous data collection in participating sites had shown that baseline levels of communication were poor and that women were not fully benefiting from health counseling by nurse-midwives during antenatal visits. Nurse-midwives were trained to use the job aids to improve their performance. Lay nurse aides were also trained to use the job aids to ensure that they had comparable performance support when assessing the effectiveness of the task shifting approach. Eleven antenatal counseling cards were organized into three modules to prioritize messages and ensure that over the course of pregnancy women would have multiple exposures to key information. These modules emphasized core messages relating to care during pregnancy, birth preparedness, danger signs, clean delivery, and newborn care (Figure 1).

A key component of task shifting is to define the task to be delegated and the training and experience needed for the type of worker to whom the shift will occur [37]. The study team separately consulted with nurse-midwives and lay nurse aides prior to the task shift. There was general consensus that the task to be delegated was communicating with women before or after the antenatal physical examination about messages relating to the health of the mother and newborn. To do so, the study team concluded that lay nurse aides would need training in use of the counseling job aids and communication skills, training in the maternal and newborn care technical content, as well as quality improvement. Task shifting also requires supervision so that it does not undermine the primary goal of improving quality of care [37]. Therefore, training also focused on competencies needed by nurse-midwives such as provision of feedback and supervision.

To prepare for the task shift, the two cadres were trained for three days separately using similar curricula. Training of both provider types included a description of the concept of task delegation, peer and group role-playing, capacity building in interpersonal communication, and emphasis on quality of care. The nurse-midwife training was conducted in French and included technical materials in French and additional instruction on planning, supervision, and evaluation. The lay nurse aide training was comparable, but provided at a slower pace and conducted in the local language, Fon, since most lay nurse aides were not proficient in written and spoken French. The courses ended with a joint session of both cadres to ensure positive intra-provider relations and confirm roles related to task shifting.

Prior to data collection, all sites received a supervisory visit from one of the trainers or technical advisors. These visits included a review of the organization of counseling using the counseling job aids, observation of consultations with direct feedback, and discussions about difficulties implementing the job aids or the task shift.

### Measurement

There were three measurement areas in the study: quality of counseling, provider perceptions of task delegation, and women's knowledge of maternal and newborn care.

To evaluate quality of counseling, providers' content of communication and counseling technique were measured through direct observation using a pre-tested observation checklist. The checklist covered five topic areas: general prenatal care, birth preparedness, dangers signs, clean delivery, and newborn care. 'General prenatal care' included four messages relating to prevention and treatment of malaria (use of an insecticide-treated mosquito net and antimalarials), iron/folate supplementation, having at least four antenatal visits, and information on diet and nutrition. 'Birth preparedness' included seven messages regarding identifying a place to deliver, identifying a skilled attendant, securing a means of transport, putting money aside, planning for emergencies, planning with a family member, and identifying a blood donor. 'Danger signs' highlighted nine maternal symptoms that require care: vaginal bleeding, convulsions, fever, water loss, abdominal pains, severe headaches, blurred vision, swelling of limbs, and absence or diminished fetal movement. 'Clean delivery' consisted of two messages relating to provision of a clean, plastic cloth for delivery and clean, dry towels for the mother and newborn. Six messages related to 'newborn care': skin-to-skin contact, early and exclusive breastfeeding, delayed bathing, clean cord care, and thermal protection. For each item, a trained observer selected 'yes' or 'no' depending on whether the woman received information regarding that item during her antenatal visit. Provider communication techniques were scored similarly across six communication techniques: presenting the subject, posing questions to determine current knowledge, using visual aid(s), verifying understanding, motivating adoption of new behaviors, and asking the woman if she has questions.

Provider perceptions on task delegation were obtained using semi-structured questionnaires during individual interviews. Following the task shift, health workers were asked whether they thought that similar approaches should be introduced in other sites, what were perceived advantages and disadvantages, and what were recommended strategies to improve task shifting. Respondents were also asked to indicate whether they agreed (indicate 'yes') or disagreed (indicate 'no') to 14 statements regarding the organization, acceptability, and effectiveness of task shifting. Responses were coded and analyzed by topic area and statement. Information on provider demographic characteristics (*e.g.*, age, education, qualification, years working in health field, years working at health center) was also obtained.

To assess maternal understanding, pregnant women were interviewed at the health center prior to departure. Structured questionnaires were written in French and administered orally in the local language. Women were asked to indicate what they considered to be important components of care during and after pregnancy for both the mother and newborn as well as what they considered to be danger signs that required urgent medical care. Women's age, months in pregnancy, education, number of previous antenatal visits, first-time visit status, and number of living children were also measured.

All data collection tools were reviewed and approved by local Beninese project staff to ensure they were clear, easy to follow, and appropriate for the local culture. The observation team received three days of training in counseling observation, interviewer techniques, and questionnaire completion, including a standardization session to minimize inter-observer variability. Pre-tested, standardized questionnaires with a detailed guide for data collectors were used with routine supervision of data collectors' instruments. Supervisors observed approximately 5% of counseling sessions and interviews for quality control purposes.

### Statistical analysis

The absence of statistical significance cannot be interpreted as equivalence [35,36]. This non-inferiority study was designed to demonstrate that the difference between the nurse-midwives and lay nurse aides is no less than the non-inferiority margin (∆_NI_) of 10%. Non-inferiority (NI) would be demonstrated if the lower confidence limit for the difference in mean percent of recommended messages between the two provider groups lay above -∆ _NI _= -10. This would mean that the null hypothesis (H_0_: ∆ >∆_NI_) is rejected in favor of the alternative hypothesis (H_A_: ∆ <∆_NI_). Any difference smaller than the lower bound would be unlikely in the population. It is important to note that the upper limit of the confidence interval (CI) is not interpreted since the study is a one-sided trial, and observed improvements are consistent with the inference of non-inferiority [34]. If the lower limit exceeds the margin on non-inferiority (∆ >∆_NI_), where the difference surpasses -10, the results are inconclusive or provide insufficient evidence to support non-inferiority (U). If the lower limit lies completely above zero, superiority is demonstrated (S).

One-sided confidence intervals of the mean percent of recommended messages provided by the two provider groups were calculated using STATA (Version 9.2, StataCorp, College Station, TX). Two sample t-tests were used to examine bivariate differences. Because data were clustered, the study employed three-level hierarchal modeling techniques to account for the inherent correlation of data given that pregnant women (level 1) were nested within providers (level 2) who were nested within sites (level 3). Random effects were modeled for provider- and site-level characteristics. Fixed effects were modeled for patient characteristics among variables that significantly varied between groups. This statistical technique is more suitable for clustered data than conventional regression analyses that underestimate standard errors by assuming observations from the same sites or providers are unrelated [38,39]. Rather, random effects hierarchal analyses aim to correct for correlation of observations and account for unmeasured differences in level-specific characteristics [40]. Random effects were used since a means-as-outcomes regression model indicated that no site or provider characteristics had significant direct effects on quality of counseling.

'Intention to treat' analyses in which patients are compared according to the assigned study arm and 'per protocol' analyses where patients are compared according to the study arm they actually received were conducted concurrently as recommended for non-inferiority studies [34,35]. The inclusion of protocol violators in intention to treat analyses increases the likelihood of finding non-inferiority since differences between groups are attenuated [34,35]. Thus, only the per protocol results are reported. Comparisons of findings between the two analytical samples were made to assess the impact of protocol violators on statistical inferences.

Maternal knowledge was analyzed using two sample tests of proportions and similar multivariate hierarchal regression to adjust for nesting of patient observations within providers and sites. Chi-squared descriptive statistics were used to calculate overall agreement with the task-shifting statements. Data were double entered using EpiData (Version 3.2) with automatic checks for range. In all analyses, the level of significance was considered at *p *≤ 0.05.

### Ethics approval

This study received ethics approval by the Johns Hopkins Bloomberg School of Public Health Institutional Review Board, Baltimore, Maryland; the Research & Evaluation review group of the USAID Health Care Improvement Project at University Research Co., LLC (URC), Bethesda, Maryland; and the USAID Integrated Family Health Project at URC, Bohicon, Benin.

## Results

### Sample characteristics

The study included 48 health care providers: 21 nurse-midwives and 27 lay nurse aides at seven sites (Table 1). Also included were 409 pregnant women: 206 who were counseled by nurse-midwives and 203 by lay nurse aides within the per protocol sample. This represented a reclassification of four pregnant women as compared to the intention to treat sample. There were no significant differences in provider characteristics. The percent of providers who had completed secondary education was lower among lay nurse aides (83%) than midwives (100%), but this was not statistically significant (p > 0.05). Mean age of nurse-midwives was 34 years compared to 35 years among lay nurse aides (p > 0.05). The average number of years spent working in public health and at the current health center was 10 and 5, respectively, among nurse-midwives and 11 and 7, respectively, among lay nurse aides (p > 0.05). All individual characteristics of women between the study groups were also comparable. Approximately half the women had less than eight years of education; mean gestational age was six months; and mean number of previous antenatal visits was three. The proportion of women who received group and individual counseling (79% and 74%) versus those who received individual counseling only (16% and 16%) were similar for nurse-midwives and lay nurse-aides, respectively.

**Table 1:** Sample characteristics for assessment of non-inferiority in antenatal counseling (per protocol)

	Nurse-midwives (n = 21)	Lay Nurse Aides (n = 27)	p-value
Study Population			
Number of sites	7	7	-
Total number of observations	206	203	-
Group and individual counseling (%)	79.1	73.9	0.15
Group counseling only (%)	4.9	9.9	
Individual counseling only (%)	16.3	16.0	
Provider characteristics			

Mean age (yrs)	33.6	35.1	0.60
Completed secondary education (%)	100	83.3	0.06
Years working in health field (yrs)	10.1	10.9	0.73
Years working at health center (yrs)	4.6	6.6	0.25
Patient characteristics			

Mean age (yrs)	25.3	25.1	0.73
Mean gestational age (months)	6.0	5.8	0.39
Educational status (%, >8 yrs)	52.4	55.9	0.48
1^st ^prenatal visit (%, in current pregnancy)	24.3	23.2	0.79
Mean number of antenatal visits (in current pregnancy)	2.7	2.7	0.99
Mean number of living children	1.5	1.5	0.79

* Significant at p < 0.05.

The proportion of women presenting at their first antenatal visit in the current pregnancy was similar in the nurse-midwife group (24%) as compared to the lay nurse aide group (23%, p > 0.05). Mean gestational age for first-time attendees was 3.9 and 3.8 months for nurse-midwives and lay nurse aides, respectively (p > 0.05). Of the observed consultations, the primary language used was similar, with 97% of counseling sessions conducted in Fon in both groups (p > 0.05). On average, there were 6.9 providers per site (nurse-midwives plus lay nurse aides) with lay nurse aides slightly out numbering nurse-midwives (ratio = 1.29) (data not shown). Approximately 58 pregnant women were observed at each site representing approximately 9.8 observed consultations per nurse-midwife and 7.5 per lay nurse aide.

### Content of communication

Table 2 presents the 95% CIs of the differences in the mean percent of recommended messages provided to pregnant women by topic and provider type. No significant differences appeared in the content of communication provided. On average, women counseled by lay nurse aides received 80% of recommended maternal and newborn care messages as compared to 75% by nurse midwives in adjusted analyses (β = 4.7, 95%CI: -1.7, 11.0; NI).

**Table 2:** Difference in mean percent of messages provided during antenatal visit, by topic and provider type (per protocol)

Mean % of messages provided	Nurse-midwives	Lay Nurse Aides	Differ-ence (β)	95% CI	**Inference**^**a**^
No. of pregnant women (N = 409)	206	203			
Adjusted Scores^b^					

Mean % of messages given (total)	75.2	79.9	4.7	-1.7, 11.0	NI
Mean % of messages given (by topic^c^)					
Prenatal care	74.6	90.3	15.7*	7.0, 24.4	S
Birth preparedness	82.9	82.9	-0.0	-9.0, 9.1	NI
Danger signs during pregnancy	68.7	73.4	4.7	-5.1, 14.6	NI
Clean delivery	87.8	89.2	1.4	-9.4, 12.3	NI
Newborn care^d^	69.0	61.7	-7.3	-23.1, 8.4	U
Mean % of communication techniques used	95.2	97.6	2.4	-0.2, 5.0	NI
Mean duration of antenatal consultation^e^	29.0	31.9	2.9	-0.7, 6.4	-

[a] Non-inferiority margin (∆) = -10 where inference drawn is designated by: S = superior; NI = non-inferior; U = insufficient evidence. [b] Scores adjusted for correlation of observations; site- and provider-level characteristics (random effects); counseling mode; and patient age, education, first prenatal visit, and total number of prenatal visits (fixed effects). [c] Total number of messages by category include: prenatal care (n = 5), birth preparedness (n = 7), danger signs during pregnancy (n = 9); clean delivery (n = 2); newborn care (n = 6); communication techniques (n = 6). [d] Includes only women at 6 - 9 months of pregnancy. [e] Excludes additional time for women who participated in individual counseling following group session. * Significant at p < 0.05. Note: Upper limits of the confidence interval are not interpreted for non-inferiority analyses.

By topic area, no significant differences in content of communication were observed in adjusted analyses between nurse-midwives and nurse aides in the area of birth preparedness, danger sign recognition, clean delivery, or newborn care. Non-inferiority was demonstrated among nurse aides for information on danger signs (β = 4.7, 95%CI: -5.1, 14.6; NI), clean delivery (β = 1.4, 95%CI: -9.4, 12.3; NI), and birth preparedness (β = -0.0, 95%CI: -9.0, 9.1; NI), but there was not sufficient evidence to demonstrate non-inferiority for messages relating to newborn care (β = -7.3, 95%CI: -23.1, 8.4; U). Nurse aides had significantly higher performance in the area of general prenatal care as compared to nurse-midwives (90% versus 75%, p < 0.05) (β = 15.7, 95%CI: 7.0, 24.4; S). In adjusted models, correlation of observations within providers and sites slightly tapered the observed unadjusted effect (not reported), although all gains remained significant. Patient characteristics did not significantly influence performance scores.

An item analysis of key messages within each topic area showed considerable variability in the proportion of women who received any one message (Table 3). For some messages such as identifying a skilled attendant and planning for birth-related emergencies, performance was significantly lower (47% and 69%, respectively) by lay nurse aides than by nurse-midwives (72% and 81%, p < 0.05). On the other hand, the item-level performance for nearly all messages within general prenatal care was significantly higher among nurse-aides, although comparable in other topic areas.

**Table 3:** Item analysis - percent of women receiving message during antenatal visit, by topic and provider type (per protocol)

	Nurse-midwives	Lay Nurse Aides	Differ-ence (β)	95% CI
No. of pregnant women (N = 409)	206	203		
Prenatal care				

Sleep under a mosquito net	74.3	90.1	15.9*	9.8, 22.0
Take anti-malarials	71.4	89.2	17.8*	11.5, 24.1
Take iron/folic supplements	75.7	90.1	14.4	8.4, 20.4
Have at least four prenatal visits	65.5	85.2	19.7*	12.9, 26.5
Eat more and more varied	73.3	86.7	13.4*	7.0, 19.8

Birth preparedness				

Identify place of delivery	85.4	84.2	-1.2	-7.0, 4.6
Identify means of transport	86.9	83.7	-3.1	-8.9, 2.6
Identify skilled attendant	71.8	46.8	-25.0*	-32.8, -17.3
Put money aside	84.5	83.7	-0.7	-6.7, 5.2
Plan for emergency	81.1	69.0	-12.1*	-19.1, -5.1
Plan with family	84.5	79.8	-4.7	-10.9, 1.6
Identify a blood donor	68.9	70.0	1.0	-6.5, 8.5

Danger signs during pregnancy				

Vaginal bleeding	71.3	75.4	4.0	-3.2, 11.2
Convulsions	53.4	43.4	-10.0*	-18.1, -2.0
Fever	71.4	73.9	2.5	-4.7, 9.8
Water loss	71.8	74.4	2.5	-4.7, 9.7
Abdominal pains	72.8	74.4	1.6	-5.6, 8.7
Severe headaches	67.5	72.9	5.4	-2.0, 12.9
Blurred vision	66.0	62.1	-3.9	-11.8, 3.8
Swelling of limbs	58.3	65.0	6.8	-1.1, 14.7
Diminished fetal movement	57.3	50.2	-7.0	-15.1, 1.1

Clean Delivery				

Bring plastic cloth	67.0	62.6	-4.4	-12.2, 3.3
Bring five clean towels	82.0	80.3	-1.7	-8.1, 4.6

Immediate newborn care^a^				

Skin-to-skin contact	45.8	53.1	7.3	-3.5, 18.0
Initiation of immediate breast feeding (BF)	57.5	56.6	-0.9	-11.5, 9.8
Avoid prelacteal foods/exclusive BF	54.2	60.2	6.0	-4.6, 16.7
Delayed bathing	41.7	45.1	3.5	-7.2, 14.1
Clean cord care	37.5	42.5	5.0	-5.6, 15.5
Thermal protection	47.5	52.2	4.7	-6.1, 15.5

Communication technique				

Presents the subject	98.5	100.0	1.5	0, 2.8
Determines woman's current knowledge	99.0	98.0	-1.0	-3.0, 0.1
Uses cards or other visual aids	99.5	100.0	0.5	-0.3, 1.3
Verifies understanding	98.5	98.5	0	-2.0, 1.9
Motivates to adapt behaviors	96.1	99.0	2.9	0.4, 5.4
Asks woman if she has questions	97.1	99.5	2.4	0.3, 4.5

[a] Includes only women at six to nine months of pregnancy. * Significant at p < 0.05

### Communication techniques and duration

Mean performance was high for both provider types with regard to communication techniques at 95% and 98% among nurse-midwives and lay nurse aides, respectively (β = 2.4, 95%CI: -0.2, 5.0; NI) (Table 2). At the item level, all communication techniques were observed in over 96% of pregnant women, suggesting widespread application of good communication skills. Total time spent in consultation was slightly higher for women counseled by lay nurse aides (32 minutes) than by nurse-midwives (29 minutes), but this was not statistically significant. It is important to note, however, that this measure does not discriminate non-communication versus communication time during antenatal consultations.

### Maternal knowledge

Although content of communication was similar between cadres, the study examined maternal knowledge following antenatal consultations to determine whether any unmeasured differences in communication, technique, or interaction between provider types influenced women's ability to understand and recall messages. Maternal knowledge among women counseling by lay nurse aides was superior in three of the five topic areas: prenatal care (β = 23.8, 95%CI: 15.7, 32.0; S), birth preparedness (β = 12.7, 95%CI: 5.2, 20.1; S), and recognition of danger signs (β = 8.6, 95%CI: 3.3, 13.9; S) (Table 4). There were no significant differences in maternal knowledge by provider type for clean delivery (β = -2.1, 95%CI: -14.1, 9.9; U) and newborn care (β = 9.9 95%CI: -0.3, 20.1; NI), although non-inferiority was demonstrated for newborn care. The mean number of correct responses by women counseled by nurse-midwives was 11.4 compared to 12.6 among women counseled by lay nurse aides (β = 1.2, 95%CI: 0.4, 1.9; p < 0.05).

**Table 4:** Differences in maternal knowledge by topic and provider type (per protocol)

Percentage (%) of women with correct responses	Nurse-midwives	Lay Nurse Aides	Difference (β) 95% CI	**Inference**^**a**^
No. pregnant women (N = 409)	206	203		
Adjusted Scores^b^				

≥3 messages in prenatal care	56.0	79.8	23.8 (15.7, 32.0)*	S
≥3 messages in birth preparedness	39.3	52.0	12.7 (5.2, 20.1)*	S
≥3 danger signs during pregnancy	76.9	85.5	8.6 (3.3, 13.9)*	S
= 2 messages in clean delivery	54.7	52.6	-2.1 (-14.1, 9.9)	U
≥3 messages in newborn care^c^	63.1	73.0	9.9 (-0.3, 20.1)	NI
Mean # correct responses	11.4	12.6	1.2 (0.4, 2.0)*	-

[a] Non-inferiority margin (∆) = -10 where inference drawn is designated by: S = superior; NI = non-inferior; U = insufficient evidence. [b] Scores adjusted for correlation of observations; site- and provider-level characteristics (random effects); counseling mode; and patient age, education, first prenatal visit, and total number of prenatal visits (fixed effects). [c] Includes only women at six to nine months of pregnancy. *Significant at p < 0.05. Note: Upper limits of the confidence interval are not interpreted for non-inferiority analyses.

### Provider perceptions

With regard to staff perceptions on the organization of task shifting, most indicated that lay nurse aides could effectively counsel pregnant women if appropriately trained and supervised (98%) and that counseling could be done by both types of providers (98%) (Table 5). However, few felt that counseling should be done by only a skilled provider (12%) or a lay nurse aide (9%). A third of providers felt that task shifting brought about some challenges (33%).

**Table 5:** Provider perceptions of task shifting using agreement statements, by type of provider

*Task-shifting Statements: *Percent (%) of providers responding 'Agree'	Nurse-midwives	Lay Nurse Aides	Total
No. of providers interviewed	n = 19	n = 24	N = 43
Holds role of counseling	Yes (prior to shift)	Yes (after shift)	
*Organization*			

The role of nurse aides can include counseling if they have the necessary support and supervision.	94.7	100.0	97.7
Counseling should only be done by skilled providers.	21.1	4.2	11.6
Counseling can be done by all maternity workers.	94.7	100.0	97.7
Counseling can be done only by nurse aides.	10.5	4.2	9.1
Task shifting is difficult and with challenges.	36.8	29.2	32.6
*Impact and Effectiveness*			

When the role of nurse aides was expanded, skilled workers had more time for clinical activities.	100.0	87.5	93.0
Quality of counseling by nurse aides is less effective than that done by skilled providers.	47.3	45.8	46.5
Quality of counseling by nurse aides is more effective than that done by skilled providers.	52.6	25.0	37.2
Task shifting of counseling to nurse aides improves provider relationships.	84.2	87.5	86.1
Shifting the role of counseling to nurse aides is more effective than the previous work organization.	89.5	83.3	86.1
*Comfort and Acceptability*			

Nurse aides are more comfortable counseling than the skilled providers.	68.4	54.2	60.5
Skilled providers are more at ease if counseling is done by nurse aides.	73.6	75.0	74.4
Counseling provided by nurse aides is accepted by women presenting at the maternity.	89.5	100.0	95.4

For statements relating to impact and effectiveness, most health workers reported that task shifting relieved skilled workers to focus on more clinical activities (93%), improved provider relationships (86%), and was more effective than the prior organization of care (86%). More than half (53%) of lay nurse aides indicated that counseling by lay nurse aides is more effective than that of nurse-midwives, who were less likely to agree (25%). Reasons for this belief were that lay nurse aides were closer to the communities, had fewer linguistic barriers, and had their training to rely on. About half of nurse aides (46%) and nurse-midwives (47%) were also concerned that lay nurse aide-led counseling is less effective because lay nurse aides needed the support of skilled providers who were more experienced in counseling and communication.

Perceptions relating to comfort and acceptability were generally positive with strong agreement that counseling provided by lay nurse aides was acceptable to women (95%). Reasons given were that women do not distinguish between the qualifications of nurse-midwives or lay nurse aides and consider all health staff to be capable of providing services. The support for lay nurse aide-led counseling being done with ease-of-mind by nurse-midwives (61%) or lay nurse aides (74%) was also high. Providers who said they were comfortable with the task shift indicated that it gave more time to nurse-midwives for clinical activities, encouraged working more efficiently in teams, and enabled communication to be provided at a high level. Those who were not comfortable with the shift raised questions about the need for counseling by lay nurse aides at instances when skilled providers had sufficient time.

Among open-ended questions, reported advantages to task shifting were that task shifting improved the continuity of services since nurse-midwives were often more occupied or likely to be absent more than lay nurse aides and that the shift clarified the role of lay nurse aides (Table 6). In particular, lay nurse aides indicated that as a result of the job aids training and their expanded role, an additional advantage was feeling more highly regarded by nurse-midwives. Reported disadvantages to task shifting were that shortages of both types of personnel in the presence of increased communication time posed challenges. Lay nurse aides suggested that having more lay nurse aides would be helpful, including improvements in supervision and support. Some nurse-midwives suggested that communication mechanisms between providers should be improved and that the role of skilled providers should still include counseling.

**Table 6:** Provider perceptions of task shifting using open-ended questions, by type of provider

**Topic area**:	*Advantages to task shifting:*	*Disadvantages to task shifting:*	***Suggestions to improve task shifting***:
**Skilled providers' responses**^a ^(n = 19^b^)	- Skilled providers have more time for clinical tasks* - Facilitates the clinical work by enabling focus on clinical tasks that reduces fatigue - Allows skilled workers to attend to urgent cases as needed* - Improves the continuity of counseling even when the skilled provider is unavailable - Requires provider confidence - Increases/expands participation of all health workers in the provision of care* - Nurse aides speak the local language(s), so decreases language barriers	- Sometimes it's possible that the counseling could be poorly done by the unskilled worker - Difficult to implement in cases where there are severe shortages of both types of providers* - Aides prolong antenatal consultation as a result of counseling	- Increase circulation of the counseling task among the nurse aides - Post delegated task items for viewing - Expand task shifting to other health centers* - Improve site-level communication between cadres - Allow skilled workers to perform counseling also

**Lay nurse aides' responses**^a ^(n = 24^c^)	- Provides more clarity on what are the tasks/role of nurse aides* - Have ability to conduct the counseling even in the absence of a skilled provider* - Women like counseling by aides - Improves the consultation - Allows aides to participate more in counseling activities - Aides received new knowledge* - Aides are more familiar/have more in common with the women from the community - Aides appreciated being promoted to new service* - Improved work relationship between providers	- Shortage of personnel makes it difficult to implement at times*	- Explore possibility of task shifting to nurse aides in other domains - Increase the number of nurse aides* - Improve supervision - Expand role of nurse aides at all sites*

[a] The symbol (*) denotes that the response was commonly stated. [b] Only 19 providers (out of 21) were interviewed. All responded 'yes' when asked if they thought task shifting should be introduced at other sites. [c] Only 24 unskilled providers (out of 27) were interviewed. All responded 'yes' when asked if they thought task shifting should be introduced at other sites.

### Operationalizing task shifting guidelines

The World Health Organization (WHO) recently released a set of recommendations for task shifting to guide programming and policy for HIV and AIDS or other health areas (17). Table 7 summarizes this study's experience in operationalizing six of the 22 recommendations according to the scope and the experimental nature of the study. The guidelines emphasize consultation and engagement of stakeholders prior to task shifting -- attributing prior unsuccessful experiences to limited involvement of appropriate parties (recommendation #4). By design, this study examined perceptions of both nurse-midwives and lay nurse aides, collaborated with Ministry Of Health representatives, and was informed by responses from women during a pilot study of use of the job aids by lay providers (unpublished data). An assessment of counseling provided to women prior to the shift suggested that informal task shifting in the counseling of pregnant women was not common (recommendation #4). Thus, the shift was introduced as a tested change within a broader quality improvement initiative (recommendation #7). Necessary competencies for effective communication in maternal and newborn care were identified and used to train and evaluate lay nurse aides (recommendation #8), with emphasis likewise on improving competencies of more skilled providers in the area of support and supervision (recommendation #11). Lay nurse aides were identified as ideal candidates given their existing employment within the public health system (recommendation #14).

**Table 7:** Selected WHO Global Recommendations for Task Shifting and related study operationalization

*Recommendation summary*^*a*^^,b^	*Study operationalization*
Endeavor to identify and involve appropriate stakeholders concerning aspects of task shifting approach (#2)	Study examined perceptions of both types of providers, including use of experience from a pilot test regarding acceptability among women.

Examine extent to which task shifting is already taking place (#4)	Study found that informal task shifting occurred primarily in absence of skilled provider and that lay nurse aides regretted lack of training. Only a small proportion of counseling was provided by lay nurse aides prior to the shift.

Adapt or create quality assurance mechanisms to support a task shifting approach that include processes and activities to monitor and improve quality of services. (#7)	The task shifting approach was adopted within a quality improvement collaborative that identifies improvement objectives and integrates site-level monitoring, coaching, and assessment of key indicators related to maternal and newborn care. Findings on effectiveness of tested changes are shared within learning sessions.

Define role and quality standards that serve as the basis for establishing recruitment, training and evaluation criteria. (#8)	Lay nurse aides were trained and evaluated based on recommended communication goals during antenatal care for pregnant women. Lay nurse aides were recruited as candidates for the task shift given their existing integration within health system and local community.

Provide supportive supervision and clinical mentoring within function of health teams that make certain that supervision staff have appropriate supervisory skills. (#11)	Task shifting approach included capacity building of nurse-midwives in supervision with emphasis on observation and feedback. Mentoring and supervision teams included technical personnel and regional trainers.

Recognize that sustainable expansion of essential health services cannot not rely on volunteer cadre. Rather, trained workers should receive adequate wages or commensurate incentives. (#14)	Lay nurse aides are paid government health staff whose wages are lower than those of nurses-midwives. Lay nurse aides reported several non-monetary incentives resulting from task shift, but efforts are needed to explore appropriate remuneration for expanded role.

[a] WHO, 2007; [b] Recommendations related to other types of task shifting, country policies, and regulatory frameworks relating to scale-up were beyond the scope of the study and not included.

## Discussion

An important criterion for task shifting is that there is no compromise in quality of care provided [37]. This study offered a non-inferiority methodology using quality assessment to determine whether antenatal counseling in maternal and newborn care provided by lay nurse aides was non-inferior to that of skilled nurse-midwives, as well as documentation of processes and perceptions to provide new insights relating to task shifting. Findings demonstrated that quality of counseling by lay nurse aides when supported by job aids is non-inferior to counseling provided by nurse-midwives with similar job supports. The study found comparable counseling performance in the area of birth preparedness, recognition of danger signs, and delivery care. In adjusted and unadjusted analyses, the lower bound of the confidence interval fell within the non-inferiority margin. Provided that lay nurse aides receive adequate training and field support, this suggests there is little reason to exclude them in efforts to improve facility-based antenatal health education.

Although non-inferior in several communication areas, lay nurse aides likewise had significantly higher mean scores in the area of general prenatal care and communication techniques without significant increases in duration of antenatal consultations as compared to nurse-midwives. We hypothesize that increases in general prenatal care may reflect lay nurse aides' increased motivation and better compliance to counseling job aid instructions. Similar conclusions were drawn in other studies [6,41]. General prenatal care may likewise have represented an information area where they were more knowledgeable. It is interesting to note, however, that informal observations suggested that some lay nurse aides were less likely to use the job aid text (written in French) on the back of the card and relied more on the images on the front of the card to conduct sessions. This is not surprising since not all lay nurse aides had strong literacy skills in French. This may explain likewise why they had slightly higher scores in the area of danger signs, for example, where there were separate images for most messages. In contrast, all messages relating to birth preparedness were not explicitly depicted in an image -- rather they were noted in text on the back of the card. This may explain lower item-level performance by lay nurse aides than nurses-midwives. However, upon review of the implications of printing text in the local language, it was concluded that overall literacy was lower in the local language than in French, so such modification may not have been helpful.

Communication by lay nurse aides was also associated with better patient outcomes as evidenced by maternal knowledge. Although content and techniques were similar for both cadres, maternal knowledge may have been higher due to lay nurse aides' better interpersonal skills [37]. It is also possible that the women paid more attention to the new cadre, enabling them to retain more. The study examined whether differences in maternal knowledge between the two cadres were explained by variations in maternal characteristics or an increase in the number of women returning from previous antenatal visits, but there was no evidence to support this.

The generally positive opinions by both types of health workers likely contributed to the intervention's success. Prior to the shift, the study team worked to garner support among stakeholders and determine perceptions of the quality of counseling. Most providers addressed this issue by talking about training and support. Thus, the task shifting approach emphasized building the capacity of those to whom the task was delegated and those who would provide supervision. This was achieved through training, role-playing, use of job aids, on-site supervision, and field support, as a well as open dialogue between providers to ensure that their specific skills were esteemed. Recent task shifting guidelines attribute use of these steps to overall effectiveness and sustainability of task shifting approaches [31].

Non-inferiority was not demonstrated in the area of newborn care, although no significant differences were observed in unadjusted and adjusted analyses. Communication regarding care of the newborn was provided only to women in advanced pregnancy. Therefore, the smaller sample size for this sub-analysis likely contributed to wider confidence intervals in the mean percent of recommended messages that limited the study's power to detect non-inferiority. As a result, wider confidence intervals may have contributed to the inconclusive results. Even so, at the item level, there were two messages for which lay nurse aides were found to be significantly inferior -- both in the area of birth preparedness. These were identifying a skilled attendant and planning for an emergency. Communication performance may have been lower for these two messages given lay nurse aides' reluctance to distinguish between qualifications of providers for delivery care (since some lay nurse aides manage deliveries in the absence of a skilled provider). In addition, the message regarding planning for emergencies had few related pictorials on the counseling job aid, which lay nurse aides tended to more rely upon.

Strengthening the pictorial and textual content of the job aid and exploring perceptions among lay nurse aides regarding selected key messages may improve performance. Efforts are needed likewise to strengthen the confidence of some lay nurse aides. The three-day training focused on all topic areas within communication in maternal and newborn care. However, one alternative may be to use a phased, stepwise approach in which less-skilled workers assume communication tasks gradually for specific topics. This would allow lay workers to gain confidence and competencies in one communication area before assuming another. Even so, training itself should not be viewed as the only strategy necessary for effective task shifting. Strong supervision is also vital. Although the study did not systematically assess supervision by nurse-midwives, anecdotal evidence suggests that supervision was not standardized, consistent, or thorough -- perhaps likewise contributing to lower confidence of lay nurse aides. How best to strengthen these processes will be integral in the effectiveness of task shifting approaches introduced at scale. This will include examining consequences of giving new supervision responsibilities to more skilled staff [27].

It is worth noting that when examining providers' perceptions, there was limited support of full delegation of the counseling role to nurse aides. Responses pointed toward 'task sharing' rather than a complete shift. This varied from the tested approach in which lay nurse aides provided counseling and nurse-midwives supervised and managed clinical care. Other studies as well have recognized that full delegation may not be possible with preference given to task inclusion approaches [6]. In this context, task shifting interventions are adapted in settings where wholly replacing the more skilled worker is not possible. Most lay nurse aides also reported that they felt there was a shortage of lay nurse aides at their health center, which may further explain why there was limited support of a full delegation of counseling. A few nurse-midwives indicated that when they were unoccupied, they would prefer the option of providing counseling to women. Such responses underline the importance of integrating task shifting strategies within a larger framework of improving management of human resources [30].

## Limitations

The limitations of this study deserve mention. This study did not use an experimental non-inferiority design in that patients were not randomized to either counseling by a nurse-midwife or lay nurse aide. Therefore, to minimize biases in which women were selectively counseled by provider type, the study had two adjacent data collection periods where sessions led by nurse-midwives were examined (prior to shift) followed by examination of sessions led by lay nurse aides (after the shift). This yielded comparable characteristics in the cohort of women observed in each group. Other limitations include the potentially conservative non-inferiority margin, an educated guess in the minimum allowable difference that was not clinically significant and that was feasible in light of available resources. Further study that includes a larger sample size and smaller margin may be warranted. In addition, the study did not assess costs associated with increased supervision to support task shifting, and there was no assessment of the quality of communication provided over time or throughout the pregnancy term. All sites were purposively selected for study participation among PISAF-supported health centers, potentially influencing the generalizability of the study's results to non-supported sites. Session observers could also not be blinded to the type of provider, which may have introduced biases, although no evidence was found of this.

### Implications

These findings have three main policy implications. One relates to regulatory frameworks needed to support training, organization, and evaluation mechanisms needed for task shifting. In Benin, the role of lay nurse aides as defined by the Ministry of Health does not include health education and counseling. Widespread use of lay nurse aides to provide counseling to pregnant women would necessitate revising current policies that not only outline appropriate tasks for the lay cadre, but also define strategies to ensure that lay nurse aides are sufficiently trained, supervised, and evaluated when assuming these tasks [30,31,37]. This study found that these supports were important for successful task delegation. Certification of competence in the new task is one strategy that should be considered as part of the task shifting initiative.

Another implication relates to the estimated costs of task shifting. Although commonly cited, savings have more recently been viewed as an unlikely benefit of task shifting and an inadequate justification for its implementation [37]. This results from increased expenses in training the less skilled cadre to assume the new role and in training the more skilled cadre to take on supervisory responsibilities. Larger expenditures may also result from the development and distribution of performance supports required for the new cadre. This may offset gross monetary gains from use of a less expensive employee. In this study, task shifting relied on these additional expenses - training, supervision, and support materials (job aids), although delegation to less skilled workers can improve coverage of essential services. More information is needed regarding the magnitude of net savings or costs incurred relative to gains in service delivery and efficiency. This will need to be taken into account in the adaptation of task shifting financing policies.

Finally, this study identified some non-monetary incentives as perceived by lay nurse aides due to the expansion in their role. Such incentives included recognition from superiors, an opportunity to be more involved in patient care, and satisfaction from an expansion of professional competencies through capacity building. Many lay nurse aides indicated that they had not received this previously. This highlights the role of incentives in the uptake of task shifting, but points likewise to policy considerations regarding payment mechanisms for less skilled cadre in sustaining task shifting approaches [17,25].

## Conclusions

This research aimed to generate evidence on the quality of antenatal counseling in maternal and newborn care by lay nurse aides as compared to nurse-midwives who traditionally assume this role. Communication by lay nurse aides with appropriate training, supervision, and job aids was found to be non-inferior to that of nurse-midwives with significant gains in maternal knowledge following antenatal consultation. This evidence along with the positive perceptions among providers is encouraging, but policy decisions should address mechanisms to appropriately regulate and monitor task shifting approaches prior to formal introduction. This includes identifying what kind of 'shift' or 'sharing' is most appropriate within broader efforts to improve management of human resources.

## Competing interests

The authors declare that they have no competing interests.

## Authors' contributions

LJ conceived and designed the study, developed the data collection instruments, supervised data collection, performed the statistical analysis, and wrote the manuscript. ASY and JA participated in the testing and finalization of the data collection instruments, conducted job aids training, participated in data collection, coordinated field implementation, reviewed the study results, and made contributions to the manuscript. MA participated in the design of the job aids and associated training, reviewed study results, and made contributions to the manuscript. AT assisted in the conception of the study, coordinated local support for research activities, and contributed to the manuscript. All authors have seen and approved the final manuscript.
